# Comparing methods to estimate treatment effects on a continuous outcome in multicentre randomized controlled trials: A simulation study

**DOI:** 10.1186/1471-2288-11-21

**Published:** 2011-02-21

**Authors:** Rong Chu, Lehana Thabane, Jinhui Ma, Anne Holbrook, Eleanor Pullenayegum, Philip James Devereaux

**Affiliations:** 1 Department of Clinical Epidemiology and Biostatistics, McMaster University, Health Sciences Centre, Room 2C7, 1200 Main Street West, Hamilton ON, L8N 3Z5, Canada; 2 Biostatistics Unit, St Joseph's Healthcare Hamilton, Hamilton ON, Canada; 3 Division of Clinical Pharmacology, Department of Medicine, McMaster University, Hamilton ON, Canada; 4 Centre for Evaluation of Medicine, St Joseph's Healthcare Hamilton, Hamilton ON, Canada

## Abstract

**Background:**

Multicentre randomized controlled trials (RCTs) routinely use randomization and analysis stratified by centre to control for differences between centres and to improve precision. No consensus has been reached on how to best analyze correlated continuous outcomes in such settings. Our objective was to investigate the properties of commonly used statistical models at various levels of clustering in the context of multicentre RCTs.

**Methods:**

Assuming no treatment by centre interaction, we compared six methods (ignoring centre effects, including centres as fixed effects, including centres as random effects, generalized estimating equation (GEE), and fixed- and random-effects centre-level analysis) to analyze continuous outcomes in multicentre RCTs using simulations over a wide spectrum of intraclass correlation (ICC) values, and varying numbers of centres and centre size. The performance of models was evaluated in terms of bias, precision, mean squared error of the point estimator of treatment effect, empirical coverage of the 95% confidence interval, and statistical power of the procedure.

**Results:**

While all methods yielded unbiased estimates of treatment effect, ignoring centres led to inflation of standard error and loss of statistical power when within centre correlation was present. Mixed-effects model was most efficient and attained nominal coverage of 95% and 90% power in almost all scenarios. Fixed-effects model was less precise when the number of centres was large and treatment allocation was subject to chance imbalance within centre. GEE approach underestimated standard error of the treatment effect when the number of centres was small. The two centre-level models led to more variable point estimates and relatively low interval coverage or statistical power depending on whether or not heterogeneity of treatment contrasts was considered in the analysis.

**Conclusions:**

All six models produced unbiased estimates of treatment effect in the context of multicentre trials. Adjusting for centre as a random intercept led to the most efficient treatment effect estimation across all simulations under the normality assumption, when there was no treatment by centre interaction.

## Background

A multicentre randomized control trial (RCT) is an experimental study "conducted according to a single protocol but at more than one site and, therefore, carried out by more than one investigator"[1]. Multicentre RCTs are usually carried out for two main reasons. First, they provide a feasible way to accrue sufficient participants to achieve reasonable statistical power to detect the effect of an experimental treatment compared with some control treatment. Second, by enrolling participants of more diverse demographics from a broader spectrum of geographical locations and various clinical settings, multicentre RCTs increase generalizability of the experimental treatment for future use [1].

Randomization is the most important feature of RCTs, for on average it balances known and unknown baseline prognostic factors between treatment groups, in addition to minimizing selection bias. Nevertheless, randomization does not guarantee complete balance of participant characteristics especially when the sample size is moderate or small. Stratification is a useful technique to guard against potential bias introduced by imbalance in key prognostic factors. In multicentre RCTs, investigators often use a stratified randomization design to achieve balance over key differences in study population (e.g. environmental, socio-economic or demographical factors) and management team (e.g. patient administration and management) at centre level to improve precision of statistical analysis [2]. Regulatory agencies recommend that stratification variables in design should usually be accounted for in analysis, unless the potential value of adjustment is questionable (e.g. very few subjects per centre) [1].

The current study was motivated by the COMPETE II trial which was designed to determine if an integrated computerized decision support system shared by primary care providers and patients could improve management of diabetes [3]. A total number of 511 patients were recruited from 46 family physician practices. Individual patients were randomized to one of the two intervention groups stratified by physician practice using permuted blocks of size 6.The number of patients treated by one physician varied from 1 to 26 (interquartiles = 7.25, 11, 15; mean = 11; standard deviation [SD] = 6). The primary outcome was a continuous variable representing the change of a 10-point process composite score based on eight diabetes-related component variables from baseline to a mean of 5.9 months' follow-up. A positive change indicated a favourable result. During the study, the possibility of clustering within physician practice and its consequence on statistical analysis was a concern to the investigators. The phenomenon of clustering emerges when outcomes observed from patients managed by the same centre, practice or physician are more similar than outcomes from different centres, practices or physicians. Clustering often arises in situations where patients are selective about which centre they belong to, patients in a centre or practice are managed according to the same clinical care paths, or patients influence each other in the same cluster [4]. Intraclass (or intracentre) correlation (ICC) is often used to quantify the average correlation between any two outcomes within the same cluster [5]. It is a number between zero and one. A large value indicates that within-cluster observations are similar relative to observations from other clusters and each observation within cluster contains less unique information. This implies that the independence assumption which many standard statistical models are based on is violated. An ICC of zero indicates that individual observations within the same clusters are uncorrelated and different clusters on average have similar observations.

Through a literature review, we identified six statistical methods that were sometimes employed to analyze continuous outcomes in multicentre RCTs: A. simple linear regression (two sample t-test), B. fixed-effects regression, C. mixed-effects regression, D. generalized estimating equations (GEE), E-1. fixed-effects centre-level analysis, and E-2. random-effects centre-level analysis. The first four methods use patient as unit of analysis, yet address centre effects differently [6-8]. Simple linear regression completely ignores centre effects that are likely to arise from two sources: (1) possible differences in environmental, socio-economic or treatment factors between centres, and (2) potential correlation among patients within centres. Although stratified randomization attempts to minimize the impact of centre on standard error of the treatment effect by ensuring that the treated and control groups are largely balanced with respect to centre, failure to control for stratification in analysis will likely inflate variance of the effect estimate. The fixed-effects model treats each participating centre as a fixed intercept to control for possible population or environmental differences among centres. This model assumes that study subjects from the same centre have independent outcomes, i.e. the intraclass correlation is fixed at zero. The mixed-effects model incorporates dependence of outcomes within a centre and treats centres as random intercepts. Proposed by Liang and Zeger [9], the generalized estimating equation (GEE) model extends generalized linear regression with continuous, categorical or count outcomes to correlated observations within cluster. Under a commonly used and perhaps oversimplified assumption, that the degree of similarity between any two outcomes from a centre is equal, an exchangeable correlation structure can be used to assess treatment effect in Model C and D. Though the within- and between-centre variances ($\sigma_{e}^{2}$ and $\sigma_{b}^{2}$) are estimated differently in these two models. Method E-1 and E-2 are routinely employed to combine information from different studies in meta-analysis [10]. One can also apply them to aggregate treatment effects over multiple centres [11-13]. The overall effect is obtained as the average within-centre effect differences over centre, using inverse-variance weighting.

To date, only a few studies have been carried out to compare the performance of statistical models in analyzing multicentre RCTs using Monte Carlo simulation [6,7,14], whereas many studies assessed the impact of ICC in cluster randomization trials. Moerbeek et al [6] compared the simple linear regression model, fixed-effects regression and fixed-effects centre-level analysis with equal centre size. Pickering et al [7] examined the bias, precision and power of three methods: simple regression, fixed-effects and mixed-effects regression assuming block randomization of size 2 or 4 on a continuous outcome. In the presence of imbalance and non-orthogonality, they found ignoring centres or incorporating them as random-effects led to greater precision and smaller type II error compared with treating centres as fixed effects. Performance of the GEE approach and centre-level methods were not investigated in that work. Jones et al [14] compared the fixed-effects and random-effects regression models to a two-step Frequentist procedure as well as a Bayesian model, in the presence of treatment by centre interaction, and recommended fixed-effects weighted method for future analysis of multicentre trials. The investigation was further expanded to assessing correlated survival outcomes from large multicentre cancer trials. A series of random-effects approaches were proposed to account for centre or treatment by centre heterogeneity in proportional hazards models [15,16].

A lack of definitive evidence on which models perform the best in various situations led to this comprehensive simulation study to examine the performance of all six commonly used models with continuous outcomes. The objective was to assess their comparative performance in terms of bias, precision (simulation standard deviation (SD) and average estimated SE), and mean squared error (MSE) of the point estimator of the treatment effect, empirical coverage of the 95% confidence interval (CI) and the empirical statistical power, over a wide spectrum of ICC value and centre size. We did not consider treatment by centre interaction this study, partly because clinicians and trialists have been making efforts to standardize the conduct and management of multicentre trials via, for instance, uniform patient selection criteria, staff training, and trial monitoring and auditing to reduce heterogeneity of treatment effects among centres. Furthermore it is uncommon to find clinical trials designed with sufficient power to detect treatment by covariate interactions.

In this paper, we survey six methods to investigate the effect of a treatment in multicentre RCTs in detail. We outline the design and analysis of an extensive simulation study, and report how model performance varies with ICC, centre size and the number of centres. We also present the estimated effect of the computer-aid decision support system on management of diabetes using different methods.

## Methods

### Approaches assessing treatment effects

We investigated six statistical approaches to evaluating effect of an experimental treatment on a continuous outcome compared with the control, for multicentre RCTs. Assuming baseline prognostic characteristics are approximately balanced between the treatment and control groups via randomization, we do not consider covariates other than centre effects in the models. The first four approaches use individual patient as unit of analysis, while centre is the unit of analysis in the last two approaches.

#### Simple linear regression (Model A)

This approach models the impact of treatment (X) on outcome (Y) via regression technique (Equation 1). In the context of a two-arm trial, this approach is the same as a two-sample t-test [6].

(1) $$Y_{ij}=\beta_{0}+\beta_{1}X_{ij}+e_{ij},$$

where *Y_ij _*is the outcome of the i-th patient in the j-th centre, *X_ij _*stands for the treatment assignment (*X_ij _*= 1 for the treatment, *X_ij _*= 0 for the control), and *e_ij _*is the random error assumed to follow a normal distribution with mean 0 and variance $\sigma_{e}^{2}$. The intercept, *β*_0 _, represents the mean outcome for the control group in all participating centres, and the slope *β*_1 _represents effect of the treatment on the mean outcome.

#### Fixed-effects regression (Model B)

This model (Equation 2) allows a separate intercept for each centre (*β*_0*j*_) as a fixed effect by restricting the scope of statistical inference to the sample of participating centres in a RCT. Interpretation for *β*_1 _remains the same as in Model A. Model A and B were fitted using the linear model procedure 'lm( )' in **R**.

(2) $$Y_{ij}=\beta_{0j}+\beta_{1}X_{ij}+e_{ij}$$

#### Mixed-effects regression (Model C)

Similar to Model B, the mixed-effects regression model assumes that the intercept *β*_0*j *_= *β*_0 _+ *b*_0*j *_follows a normal distribution N(*β*_0_, $\sigma_{b}^{2}$), and is thus random effect. In Equation 3, *b*_0*j *_is the random deviation from the mean intercept *β*_0_, specific for each centre.

(3) $$Y_{ij}=\beta_{0}+b_{0j}+\beta_{1}X_{ij}+e_{ij}$$

Similar to the previous models, the within-centre variability is reflected by $\sigma_{e}^{2}$. The variability of outcome between-centre is captured by $\sigma_{b}^{2}$ in Model C. The variance and covariance of outcomes in the same or different centres can be expressed as: $\text{Var}(Y_{ij})=\sigma_{b}^{2}+\sigma_{e}^{2}$, $\text{Cov}(Y_{ij},Y_{i'j})=\sigma_{b}^{2}$, $\text{Cov}(Y_{ij},Y_{i'j'})=0$. The intraclass correlation that measures the correlation among outcomes within centre is given by $\frac{\sigma_{b}^{2}}{\sigma_{e}^{2}+\sigma_{b}^{2}}$, assumed equal across all centres. We fitted Model C in **R **via linear mixed-effects procedure 'lme( )' using the restricted maximum likelihood (REML) method [17,18].

#### Generalized estimating equations (Model D)

The GEE method has gained increasing popularity among health science researchers for its availability in most statistical software. As opposed to the mixed-effects method that estimates treatment difference between arms and individual centre effects, the GEE approach models the marginal population-average treatment effects in two steps: 1) it fits a naïve linear regression assuming independence between observations within and across centres, and 2) it estimates parameters of the working correlation matrix using residuals in the naïve model and refit regression model to adjust standard error and confidence interval for within-centre dependence [19]. As a result, the estimated impact of treatment on the outcome in GEE model reflects the "combined" within- and between-centre relationship. GEE employs quasi-likelihood to estimate regression coefficients iteratively, and a working correlation needs to be supplied to approximate the within centre correlation. When the working correlation is mis-specified, the sandwich-based covariance estimator will lead to a robust yet less efficient estimate of treatment effect in GEE model [9]. Recently, statisticians found that variance of the estimated treatment effect could be underestimated when the number of centres was small [20]. We therefore assessed the efficiency of GEE models using procedure 'gee( )' in library(gee) in **R**. As in the mixed-effects model, an exchangeable correlation structure was assumed in fitting Model D.

#### Centre-level fixed-effects model (Model E - 1)

**Figure 1:**
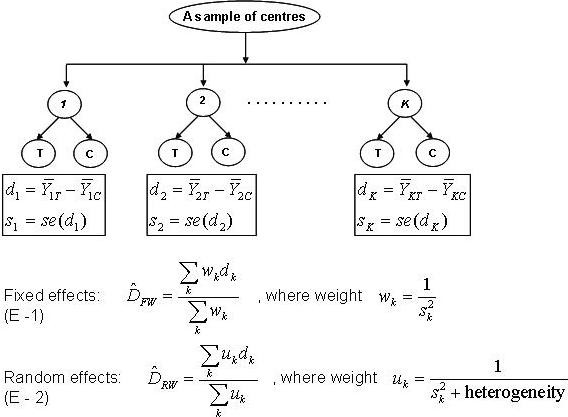
**A schematic of fixed- and random-effects centre-level models**.

The centre level model is a stratified analysis performed on the mean difference in outcome between the treatment and control arms within centre. The overall treatment effect is estimated by a weighted average of individual mean differences across all centres. The principle of inverse-variance weighting is often used (Figure 1). This model is essentially a centre-level inverse-variance weighted paired t-test (i.e. the treatment arm is paired to the control arm in the same centre) to account for within centre correlation [10]. In the absence of intraclass correlation and under the assumption of equal sampling variation at patient level, the inverse-variance weight reduces to $\frac{n_{tj}n_{cj}}{n_{tj}+n_{cj}}$ for the j-th centre, which can be further simplified as the size of centre *n_j _*= *n_tj _*+ *n_cj_*, given equal numbers of patients in two arms. Here *n_tj _*and *n_cj _*represent the number of patients in the treatment and control group, respectively, in the j-th centre. This form of the weighted analysis (without adjustment for covariates) was discussed extensively by many researchers [21-23]. We implemented Models E - 1 using the fixed-effects method for meta-analysis provided by the 'metacont( )' procedure in **R**.

#### Centre-level random-effects model (Model E - 2)

A random-effects approach is used to aggregate mean effect differences not only across all participating centres but also across a population of centres represented by the sample. This model factors heterogeneity of treatment effect among centres (i.e. random treatment by centre interaction) into its weighting scheme and captures within- and between-centre variation of the outcome. One should not confuse this method with the mixed-effects model using patient-level data (Model C). For Model E-2, the underlying true treatment effects are not a fixed single value for all centres, rather they are considered random effects, normally distributed around a mean treatment effect with between-centre variation. Model C, on the other hand, treats centres as random intercepts and postulates the same treatment effect across all centres. Model E-2 does not serve as a fair comparator to the alternatives listed here which assume no treatment by centre interaction. Preliminary investigation suggested E-2 could outperform E-1 in some situations; we therefore included E-2 in the study to advance understanding of these models. Details of centre level models are provided in Figure 1. Model E - 2 was carried out using DerSimonian-Laird random-effects [24] method using the 'metacont( )' procedure in **R**. The confidence interval for Model E - 2 was constructed based on the within- and between-centre variances.

### Study data simulation

We used Monte Carlo simulation to assess performance of statistical models to analyze parallel group multicentre RCTs with a continuous outcome. We simulated outcome, *Y*, using the mixed-effects linear regression model (Model C): *Y_ij _*= *β*_0 _+ *b*_0*j *_+ *β*_1_*X_ij _*+ *e_ij _*for the i-th patient in the j-th centre, where *X_ij_*(= 0, 1) is the dummy variable for treatment allocation (i = 1...*m_j_*, j = 1... J). We generated random error, *e*, from $N(0,\sigma_{e}^{2}=1)$. We set the true treatment effect (*β*_1_) to be 0.5 residual standard deviation (*σ_e_*), an effect size suggested by the COMPETE II trial. This corresponds to a medium effect size according to Cohen's criterion [25]. To simulate centre effects, we employed the relationship between ICC and $\sigma_{b}^{2}:\text{ICC}=\frac{\sigma_{b}^{2}}{\sigma_{e}^{2}+\sigma_{b}^{2}}$: To fully study the behaviour of candidate models at various ICC levels, we considered the following values of ICC for completeness: 0.00, 0.01, 0.05, 0.10, 0.15, 0.20, 0.25, 0.30, 0.35, 0.40, 0.45, 0.50 and 0.75. This in turn set the corresponding $\sigma_{b}^{2}$ values to be 0, 1/99, 1/19, 1/9, 3/17, 1/4, 1/3, 3/7, 7/13, 2/3, 9/11, 1 and 3. However, we focused interpretation of the results on lower values of ICC as they were more likely to occur in practice [26-28].

The original sample size was determined to be 84 per arm using a two-sided two-sample t-test (Model A) to ensure 90% power to detect a standardized effect size of 0.5 at 5% type I error rate. We increased the final sample size to 90 (Power increases to 91.8%) per arm to accommodate more combinations of the number and size of participating centres. We assumed patients were randomly allocated to two groups with a ratio of 1:1, the most common and efficient choice. We generated data in nine scenarios (Table 1) to assess model performance in three designs: (a) balanced studies where equal numbers of patients are enrolled from study centres and the numbers of patients in the two arms are the same (fixed by design); (b) unbalanced studies where equal numbers of patients are enrolled from study centres but the numbers of patients in two arms within centre may be different due to chance yet remain 1:1 allocation ratio; and (c) unbalanced studies where the numbers of patients enrolled vary among centres, and block randomization of size 2 or 4 is used to reduce chance imbalance. For designs (a) and (b), we considered three combinations of centre size and number of centres: J = 45 centres, 4 patients per centre; J = 18 centres, 10 patients per centre; and J = 6 centres, 30 patients per centre. Design (c) mimicked a more realistic scenario for multicentre RCTs. For the first setup of design (c), we grouped 180 patients to 17 centres. It was constructed so that the centre composition and degree of allocation imbalance were analogous to the COMPETE II trial but at a smaller sample size: the number of patients per centre varying from 1 to 28; quartiles = 5, 10, 15; mean = 11; SD = 8; percentage of unbalanced centres between 47% and 70% depending on block size.

**Table 1:** Catalogue of simulation designs

Design	Scenario	Number of centres	Centre size	ICC
Balance	1	45	4	
		
	2	18	10	
		
	3	6	30	
	
Chance imbalance	4	45	4	
		
	5	18	10	0 - 0.75
		
	6	6	30	
	
Blocking (size = 2)	7	17	1, 1, 4, 5, 5, 5, 8, 8, 10, 10, 10	
		
Blocking (size = 4)	8	17	10, 15, 15, 20, 25, 28	

Blocking (size = 6)	9	46	Same as Table 2	0.125

ICC: Intraclass (intracentre) correlation

To compare results from various models in analyzing the COMPETE II trial, and assess accuracy and precision of the effect estimates, we included an additional scenario in design (c) to imitate this motivating example more closely, with respect to sample size and centre composition (scenario 9). We generated treatment allocation (X1) and outcome (Y) for 511 patients in 46 centres, where the number of patients per centre was set exactly the same as observed in the COMPETE II trial (Table 2). In particular, three centres recruiting only one patient was simulated. Analogously to COMPETE II, a fixed block size of 6 was used to assign patients to treatments. The same simulation model was employed as in previous scenarios yet a separate set of parameters based on results of the COMPETE II trial were used (Table 3): *β*_0 _= 1.34, *β*_1_= 1.26, $\sigma_{b}^{2}=1$, $\sigma_{e}^{2}=7$, ICC = 0.125.

**Table 2:** Centre composition of the COMPETE II trial

Number of patients per centre	Number of Centres
1	3

2	0

3	1

4	4

5	1

6	1

7	2

8	3

9	4

10	3

11	5

12	3

13	2

14	0

15	3

16	3

17	0

18	2

19	2

20	1

21	0

22	1

23	1

24	0

25	1

**Table 3:** Estimates of intervention effects in COMPETE II trial

Model	Estimate of intervention effect	SE	95% CI	Variance component
A: Simple linear regression	1.270	0.246	(0.787, 1.753)	$\sigma_{e}^{2}=7.712$

B: Fixed-effects regression	1.291	0.231	(0.836, 1.745)	$\sigma_{e}^{2}=6.682$

C: Mixed-effects regression	1.263	0.230	(0.811, 1.714)	$\sigma_{e}^{2}=6.678$ $\sigma_{b}^{2}=1.069$

D: GEE	1.263	0.193	(0.884, 1.641)	

E - 1: centre-level Fixed-effects model	1.397	0.219	(0.967, 1.826)	

E - 2: centre-level Random-effects model	1.397	0.219	(0.967, 1.826)	

SE: standard error; CI: confidence interval; $\sigma_{e}^{2}$: within-centre variance; $\sigma_{b}^{2}$: between-centre variance; ICC: intraclass (intracentre) correlation

We generated 1000 simulations for each of the 13 ICC values under each of the first eight scenarios and 1000 simulations for the specified ICC value for the ninth scenario. Separate sets of centre effects were simulated for each scenario and each simulation 1-1000. We chose to simulate 1000 replicates so that the simulation standard deviation for the empirical power at a nominal level of 90% in the absence of clustering was controlled at 1%. This also ensured that standard deviations of the coverage of the confidence interval and the empirical power not exceed 1.6%.

### Comparison of analytic models

We applied six statistical models to each simulated dataset. For each model, we calculated the bias, simulation standard deviation (SD), average of estimated standard error (SE) and mean squared error (MSE) of the point estimator of treatment effect (i.e. *β*_1_), empirical coverage of the 95% confidence interval around *β*_1 _and the empirical statistical power. We constructed confidence intervals based on t-test for Models A - C, and Wald interval based on normal approximation for Models D and E. We estimated bias as the difference between the average estimate of *β*_1 _over 1000 simulated datasets and the true effect. The simulation or empirical SD was calculated as the standard deviation of the estimated *β*_1_s across simulations, indicating precision of the estimator. We also obtain average of the estimated SEs from 1000 simulations to assess accuracy of variance estimator from each simulation dataset. The overall error rate of the point estimator was captured by the estimated MSE, enumerated by the average squared difference between the estimated *β*_1 _and true value across the 1000 datasets. Furthermore, we reported performance of the interval estimators in each model. The empirical coverage was estimated as the proportion of 95% confidence intervals that covered the true *β*_1_, and the empirical power was the proportion of confidence intervals that rejected a false null hypothesis, i.e. zero lies outside CI. All datasets were simulated and analyzed in **R **version 2.4.1[29].

## Results

### Analysis of COMPETE II trial data

We applied all six models to the COMPETE II data and reported results in Table 3. Approximately equal numbers of patients were randomized to the intervention and control groups within each family doctor, leading to 253 and 258 patients in the intervention and control group, respectively. Among 46 family physicians, 11 physicians (24%) treated equal numbers of patients in two arms, 24 physicians (52%) treated one more patient in the intervention or control arm, 10 physicians (22%) managed 2 more patients in either arm, and one physician (2%) managed 3 more patients in one arm compared with the other.

All baseline characteristics were roughly balanced between arms [3]. The analyses using patient-level data produced similar estimates for *β*_1 _and the effect size was around 0.5 times the corresponding residual standard deviation. The standard error of the estimated *β*_1 _reduced from 0.25 (Model A) to 0.23 (Models B, C) then 0.19 (Model D) when centre effects were adjusted, leading to narrower CIs around estimated *β*_1 _in Models B - D. The intraclass correlation was estimated 0.138 in Model C and 0.124 in Model D. The two centre-level analyses returned slightly larger estimates of *β*_1 _than those from the individual patient-level models. In fact the minimal variance between physicians indicated no noticeable heterogeneity between physicians (τ^2 ^= 0, *I*^2 ^= 0), resulting in same estimates from E-1 and E-2. Zero was not contained in the 95% confidence intervals, therefore all models led to the conclusion that the experimental intervention significantly improved patient management over usual care based on the change of composite process score.

### Balanced design with equal centre size

#### Properties of point estimates

Table 4 summarizes descriptive statistics of the point estimator of treatment effect in Models A - E for three values in the lower range of the spectrum of ICC, in the balanced design. The point estimates of *β*_1 _were unbiased in all six models for all ICC values. Upon review, it was surprising that the point estimates in Model A, ignoring stratification and clustering, were invariant of ICC, and that the same estimates were returned by four patient-level models for each simulation. In fact, when treatments are allocated in same proportion in all centres, centre has no association with the treatment allocation, hence adjusting for centre effect or not has little impact on point estimate of the treatment - response relationship given a continuous response variable. For this reason, different ways to incorporate between-centre information (Models B-D) led to same estimates of treatment contrast in a balanced design. Same point estimates led to same empirical SD and overall error rate (measured by MSE) of the estimator in Models A - D regardless of ICC. Across different ICC values and scenarios 1 - 3, Models B and C yielded accurate estimates of the standard error of ${\hat{\beta }}_{1}$ that approximated the empirical SD and the true standard deviation, 0.149, calculated using the best linear unbiased estimator of the simulation model, i.e. Model C [18]. From Table 4 we found that the standard error of ${\hat{\beta }}_{1}$, in Model A increased with ICC in each scenario, deviating from the corresponding empirical SD. The standard error could be slightly underestimated in Model D when the number of centres was small (Table 4 scenario 2 and 3 comparing empirical SD and average SE). This agreed with previous work concerning small sample properties of the GEE model [20].

**Table 4:** Properties of point estimates of the treatment effect from Models A - E in scenarios 1 to 3

	ICC = 0.01			ICC = 0.05			ICC = 0.20		
**Model**	**Mean** **effect** **(SD)**	**Ave**. **SE**	**MSE**	**Mean** **effect** **(SD)**	**Ave**. **SE**	**MSE**	**Mean** **effect** **(SD)**	**Ave**. **SE**	**MSE**

Scenario 1 - balanced design, 45 centres each with 4 subjects									

A	0.496 (0.148)	0.149	0.022	0.499 (0.146)	0.152	0.021	0.497 (0.151)	0.167	0.023

B	0.496 (0.148)	0.148	0.022	0.499 (0.146)	0.148	0.021	0.497 (0.151)	0.149	0.023

C	0.496 (0.148)	0.147	0.022	0.499 (0.146)	0.148	0.021	0.497 (0.151)	0.149	0.023

D	0.496 (0.148)	0.146	0.022	0.499 (0.146)	0.146	0.021	0.497 (0.151)	0.147	0.023

E-1	0.496 (0.494)	0.066	0.244	0.491 (0.454)	0.066	0.206	0.506 (0.447)	0.065	0.200

E-2	0.499 (0.163)	0.172	0.027	0.497 (0.166)	0.170	0.027	0.494 (0.162)	0.170	0.026

Scenario 2 - balanced design, 18 centres each with 10 subjects									

A	0.490 (0.149)	0.150	0.022	0.504 (0.155)	0.152	0.024	0.498 (0.145)	0.166	0.021

B	0.490 (0.149)	0.149	0.022	0.504 (0.155)	0.149	0.024	0.498 (0.145)	0.149	0.021

C	0.490 (0.149)	0.148	0.022	0.504 (0.155)	0.148	0.024	0.498 (0.145)	0.149	0.021

D	0.490 (0.149)	0.142	0.022	0.504 (0.155)	0.143	0.024	0.498 (0.145)	0.142	0.021

E-1	0.490 (0.178)	0.130	0.032	0.501 (0.180)	0.130	0.032	0.498 (0.171)	0.130	0.029

E-2	0.492 (0.164)	0.154	0.027	0.503 (0.165)	0.155	0.027	0.498 (0.158)	0.153	0.025

Scenario 3 - balanced design, 6 centres each with 30 subjects									

A	0.496 (0.149)	0.149	0.022	0.492 (0.149)	0.152	0.022	0.504 (0.151)	0.164	0.023

B	0.496 (0.149)	0.149	0.022	0.492 (0.149)	0.149	0.022	0.504 (0.151)	0.149	0.023

C	0.496 (0.149)	0.149	0.022	0.492 (0.149)	0.149	0.022	0.504 (0.151)	0.149	0.023

D	0.496 (0.149)	0.130	0.022	0.492 (0.149)	0.130	0.022	0.504 (0.151)	0.149	0.023

E-1	0.497 (0.153)	0.144	0.023	0.491 (0.154)	0.144	0.024	0.508 (0.156)	0.144	0.024

E-2	0.497 (0.151)	0.163	0.023	0.491 (0.151)	0.163	0.023	0.507 (0.153)	0.161	0.023

SD: empirical standard deviation; Ave. SE: average estimated SE; MSE: mean squared error; ICC: intraclass (intracentre) correlation

The centre-level analyses produced larger empirical SE and MSE for ${\hat{\beta }}_{1}$ compared with the patient-level analyses given small or moderate centre sizes (Table 4). The difference reduced as centre size increased. When only a few patients were enrolled per centre, the fixed-effects centre-level point estimator in Model E - 1 had large sampling variation that was severely underestimated at all ICC values. The random-effects model (E - 2) based on DerSimonian-Laird method on the other hand seemed to yield valid SE for ${\hat{\beta }}_{1}$ that was on average greater than SEs from the patient-level models. The average estimate of SE for ${\hat{\beta }}_{1}$ over all simulations in Model E - 2 was always larger than estimates of SE in Models B and C, followed by the SE estimated in Model E - 1 across different combinations of centre size and number of centres. In this study, although datasets were generated so that the treatment effects were homogeneous among centres (i.e. no treatment by centre interaction), random-effects analysis using centre-level data outperformed the fixed-effects analysis when the centre size was small, for Model E - 2 took into account the observed "heterogeneity" due to imprecise estimation of the centre mean difference and the associated standard error.

#### Properties of interval estimates

The empirical coverage of confidence intervals (CIs) and the statistical power in balanced studies are displayed in Table 5. Models B and C produced similar coverage close to the nominal value of 95% over different ICC values and centre composition. Model A provided conservatively high coverage increasing with ICC, illustrating that for moderate to large ICC values, CIs in Model A were abnormally wide due to overestimated SE for ${\hat{\beta }}_{1}$. The empirical coverage of CIs from Model D or E - 1 on average was farther down from 95% compared with Models B and C. This is likely caused by underestimation of the standard error in Models D and E-1, and is associated with an apparent increase of power in the first three scenarios. For Model D, the coverage dropped to below 90% when the number of centres reduced to six in scenario 3. The coverage of Model E - 1 was too low to be useful when studies were conducted at many smaller centres (scenario 1). However, coverage increased gradually with centre size and approached 95% when there were 30 patients per centre (scenario 3). Model E-2 presented similar coverage pattern to E-1, although the coverage was closer to 95%. Models B and C largely maintained nominal power of 91.8% regardless of ICC value. Power of Model A decreased dramatically as ICC departed from 0, indicating that the model failed to adjust for between-centre variation or within-centre correlation in the outcome measure. The nominal type II error rate (8%) was maintained in Models D and E - 1 in scenarios 1 - 3. Model E - 2 generally had lower power to detect the true treatment effect due to a larger standard error that reflects both the within-centre variability and treatment by centre interaction. Interestingly, this power rose as the number of centres reduced and approached 88% in scenario 3.

**Table 5:** Coverage of the 95% interval estimate of the treatment effect and statistical power of Models A - E in scenarios 1 to 3

	ICC = 0.01		ICC = 0.05		ICC = 0.20	
**Model**	**Cover**. **of CI**	**Power**	**Cover**. **of CI**	**Power**	**Cover**. **of CI**	**Power**

Scenario 1 - balanced design, 45 centres each with 4 subjects						

A	0.952	0.901	0.953	0.912	0.973	0.862

B	0.947	0.905	0.945	0.924	0.951	0.899

C	0.947	0.907	0.944	0.924	0.951	0.899

D	0.941	0.911	0.936	0.931	0.933	0.902

E-1	0.286	0.902	0.294	0.920	0.320	0.912

E-2	0.933	0.810	0.921	0.818	0.938	0.821

Scenario 2 - balanced design, 18 centres each with 10 subjects						

A	0.955	0.899	0.941	0.903	0.973	0.881

B	0.954	0.906	0.935	0.906	0.954	0.916

C	0.951	0.908	0.935	0.906	0.954	0.916

D	0.929	0.909	0.902	0.919	0.940	0.924

E-1	0.845	0.904	0.835	0.917	0.857	0.924

E-2	0.921	0.868	0.905	0.886	0.938	0.875

Scenario 3 - balanced design, 6 centres each with 30 subjects						

A	0.953	0.905	0.949	0.901	0.966	0.888

B	0.948	0.907	0.947	0.906	0.952	0.918

C	0.948	0.907	0.946	0.906	0.952	0.918

D	0.860	0.915	0.854	0.931	0.867	0.929

E-1	0.939	0.918	0.931	0.910	0.926	0.927

E-2	0.952	0.867	0.949	0.846	0.953	0.880

Cover. of CI: coverage proportion of 95% confidence interval; ICC: intraclass (intracentre) correlation

Overall, Models B and C had very close performance that outweighed other models in balanced design. Models C and D converged to a solution in all simulations.

### Design with equal centre size and chance imbalance

#### Properties of point estimates

Performance of different models in multicentre studies of equal centre sizes, 1-to-1 allocation ratio and chance imbalance is displayed in Table 6 and 7. Similar results were observed as in the balanced design, though a few differences emerged. The unbalanced allocation of patients into treatment arms due to pure within-centre variation introduced chance imbalance (in both directions) into treatment - response relationship, hence ignoring centre effects completely (as in Model A) led to unbiased yet less efficient estimates for large ICC values. Model B could be less precise than Model A given small to moderate ICC values, a phenomenon previously reported by Pickering and Weatherall [7]. As in the balanced design, the fixed- and random-effects models performed comparably for various ICC values, largely because the fixed and random intercepts for study centres cancelled out in estimating effect contrast when we fit Models B and C, and had little impact on the estimation of the fixed effect contrast across centres. However, the fixed-effects model produced larger empirical standard deviation and average standard error in scenario 4, a study being composed of many centres each managing a few patients. Adjusting for between-centre variation as random effects in Model C or using population-averaged analysis in Model D allowed to borrow information across centres and resulted in greater precision.

**Table 6:** Properties of point estimates of the treatment effect from Models A - E in scenarios 4 to 6

	ICC = 0.01			ICC = 0.05			ICC = 0.20		
**Model**	**Mean** **effect** **(SD)**	**Ave**. **SE**	**MSE**	**Mean** **effect** **(SD)**	**Ave**. **SE**	**MSE**	**Mean** **effect** **(SD)**	**Ave**. **SE**	**MSE**

Scenario 4 - chance imbalance, 45 centres each with 4 subjects									

A	0.502 (0.146)	0.150	0.021	0.511 (0.154)	0.154	0.024	0.494 (0.168)	0.166	0.028

B	0.506 (0.162)	0.172	0.026	0.510 (0.174)	0.172	0.030	0.496 (0.180)	0.172	0.032

C	0.502 (0.145)	0.149	0.021	0.511 (0.155)	0.152	0.024	0.496 (0.165)	0.159	0.027

D	0.501 (0.146)	0.146	0.021	0.511 (0.155)	0.149	0.024	0.496 (0.165)	0.155	0.027

E-1	0.492 (0.525)	0.122	0.275	0.504 (0.544)	0.126	0.296	0.481 (0.482)	0.127	0.232

E-2	0.506 (0.274)	0.265	0.075	0.515 (0.284)	0.269	0.081	0.490 (0.285)	0.260	0.081

Scenario 5 - chance imbalance, 18 centres each with 10 subjects									

A	0.495 (0.148)	0.150	0.022	0.498 (0.150)	0.153	0.023	0.497 (0.169)	0.166	0.028

B	0.494 (0.156)	0.157	0.024	0.498 (0.152)	0.157	0.023	0.500 (0.161)	0.157	0.026

C	0.495 (0.148)	0.150	0.022	0.498 (0.149)	0.151	0.022	0.499 (0.159)	0.153	0.025

D	0.494 (0.148)	0.142	0.022	0.498 (0.150)	0.144	0.022	0.499 (0.159)	0.148	0.025

E-1	0.488 (0.206)	0.130	0.042	0.498 (0.199)	0.130	0.039	0.503 (0.204)	0.130	0.042

E-2	0.490 (0.177)	0.163	0.031	0.501 (0.172)	0.162	0.030	0.501 (0.178)	0.164	0.032

Scenario 6 - chance imbalance, 6 centres each with 30 subjects									

A	0.499 (0.153)	0.149	0.023	0.502 (0.150)	0.153	0.022	0.510 (0.165)	0.164	0.027

B	0.499 (0.155)	0.150	0.024	0.501 (0.150)	0.151	0.022	0.507 (0.151)	0.152	0.023

C	0.499 (0.153)	0.149	0.024	0.503 (0.149)	0.150	0.022	0.507 (0.151)	0.151	0.023

D	0.498 (0.154)	0.129	0.024	0.503 (0.150)	0.131	0.022	0.508 (0.151)	0.134	0.023

E-1	0.498 (0.159)	0.146	0.025	0.502 (0.156)	0.146	0.024	0.507 (0.157)	0.146	0.025

E-2	0.498 (0.157)	0.165	0.025	0.502 (0.153)	0.164	0.023	0.507 (0.154)	0.167	0.024

SD: empirical standard deviation; Ave. SE: average estimated SE; MSE: mean squared error; ICC: intraclass (intracentre) correlation

**Table 7:** Coverage of the 95% interval estimate of the treatment effect and statistical power of Models A - E in scenarios 4 to 6

	ICC = 0.01		ICC = 0.05		ICC = 0.20	
**Model**	**Cover**. **of CI**	**Power**	**Cover**. **of CI**	**Power**	**Cover**. **of CI**	**Power**

Scenario 4 - chance imbalance, 45 centres each with 4 subjects						

A	0.954	0.910	0.949	0.918	0.942	0.845

B	0.966	0.846	0.946	0.822	0.931	0.810

C	0.954	0.912	0.945	0.917	0.934	0.870

D	0.948	0.924	0.934	0.926	0.934	0.878

E-1	0.411	0.782	0.417	0.793	0.424	0.745

E-2	0.897	0.468	0.900	0.501	0.887	0.468

Scenario 5 - chance imbalance, 18 centres each with 10 subjects						

A	0.954	0.898	0.949	0.900	0.942	0.843

B	0.946	0.874	0.959	0.891	0.937	0.891

C	0.952	0.898	0.951	0.904	0.939	0.895

D	0.922	0.916	0.932	0.911	0.910	0.900

E-1	0.776	0.868	0.794	0.890	0.791	0.895

E-2	0.905	0.810	0.918	0.834	0.918	0.839

Scenario 6 - chance imbalance, 6 centres each with 30 subjects						

A	0.950	0.897	0.953	0.905	0.961	0.860

B	0.949	0.892	0.954	0.907	0.961	0.916

C	0.950	0.897	0.952	0.905	0.959	0.910

D	0.856	0.911	0.879	0.918	0.874	0.908

E-1	0.922	0.904	0.931	0.921	0.931	0.913

E-2	0.944	0.831	0.951	0.867	0.955	0.857

Cover. of CI: coverage proportion of 95% confidence interval; ICC: intraclass (intracentre) correlation

#### Properties of interval estimates

Similar results were observed relative to the balanced design. Patient - level models A - C guaranteed nominal coverage of confidence intervals at different ICC values, whereas the other models were likely to produce lower coverage under certain conditions. Among all models, Models C and D achieved the best empirical power that was closest to the nominal value of 91.8% across different centre sizes. When centre size was small and the number of centre was large (scenario 4), power for Models C and D also decreased with ICC, a pattern that was less obvious in scenarios 5 and 6. Models C and D achieved convergence in analyzing all simulated datasets.

### Design with unequal centre sizes and chance imbalance

**Figure 2:**
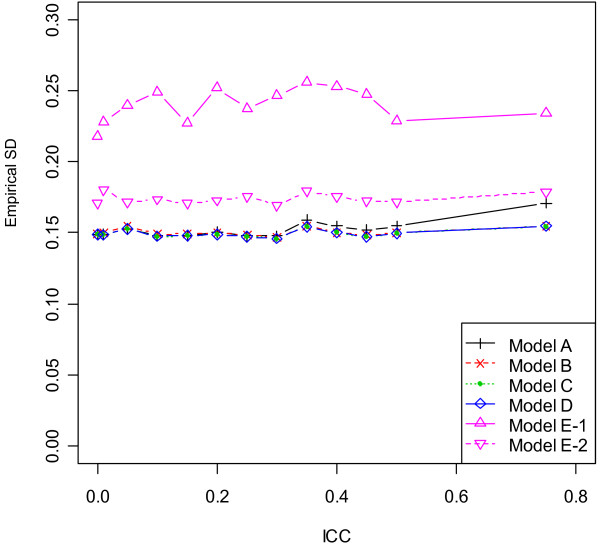
**Empirical standard deviation (SD) across 1000 simulations by ICC for scenario 8 (block size = 4)**.

**Figure 3:**
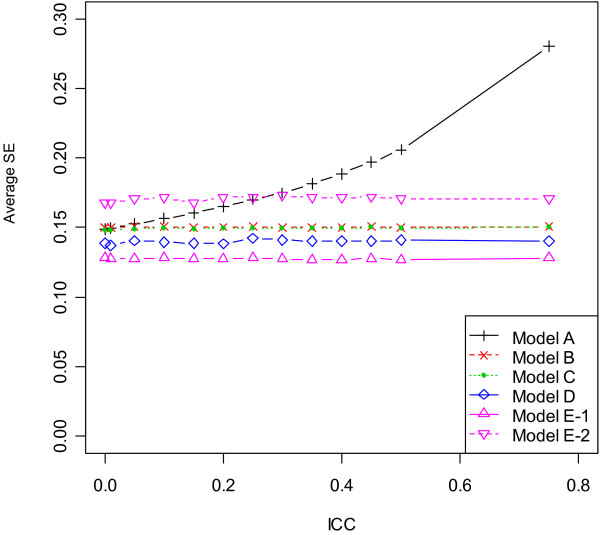
**Average of standard error (SE) across 1000 simulations by ICC for scenario 8 (block size = 4)**.

**Figure 4:**
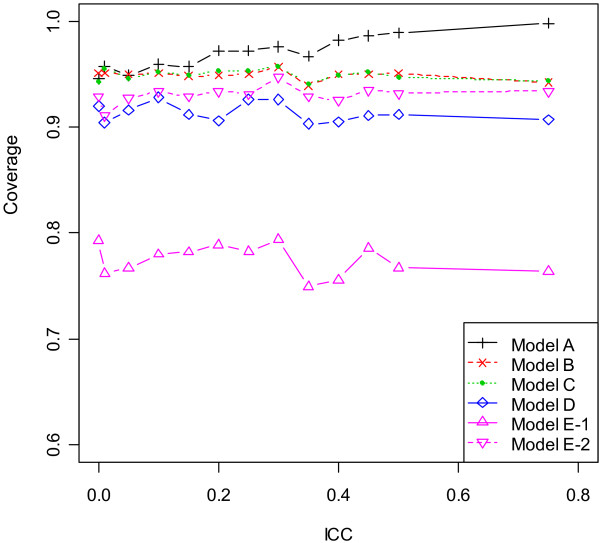
**Coverage of 95% CI by ICC for scenario 8 (block size = 4)**.

**Figure 5:**
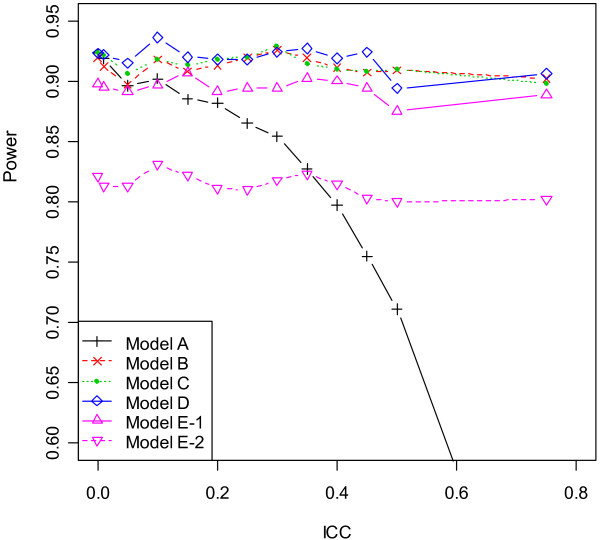
**Empirical power by ICC for scenario 8 (block size = 4)**.

The properties of point and interval estimates in the scenarios 7 and 8 (with unequal centre sizes and chance imbalance) were close to results in the previous two designs. In particular, the comparative performance of six models lay in the middle ground between scenarios 2 and 5, as the level of imbalance between two treatments was no more than half of the block size within centres. As similar results were observed for block sizes 2 and 4, summary statistics based on block size 4 were plotted in Figure 2, 3, 4 and 5. Results suggested that unequal centre size had little impact on model performance, yet it was associated with slight enlargement of the empirical variance of ${\hat{\beta }}_{1}$ in Model E - 1. To summarize, although all six models produced unbiased point estimates, the fixed- and mixed-effects models using patient-level data provided the most accurate estimates of the standard error of ${\hat{\beta }}_{1}$ given large ICC values, hence should be used in the analysis of multicentre trials when the ICC was nontrivial or unknown to control type I and type II error rates. For studies consisting of a large number of centres with only a few patients per centre, adjusting for centre as mixed effects produced most precise point estimate of treatment effect, hence were more preferable. The information sandwich method appeared to slightly underestimate the actual variance when patients were recruited from 17 centres in scenarios 7 or 8. Due to varying centre sizes, Model D did not converge for all simulated datasets (number varied between 1 and 93 out of 1000 simulations) after 2000 iterations, when ICC was less than or equal to 0.1 or greater than 0.4 for block size of 2 or 4. Such datasets were excluded for all models and extra data were simulated to attain a total number of 1000 simulations for any ICC value. In most cases, the non-convergence of GEE occurred due to a non-positive definite working correlation matrix.

In scenario 9, as a result of mimicking the particular centre composition of the COMPETE II trial, on average, three centres out of 46 contained no patients in one of the treatment groups per simulation. These centres were removed from the fixed-effects model (Model B), as no comparison patients in the same centre were available. About six centres out of 46 recruited less than two patients per treatment arm for each simulation. These centres were dropped from the centre-level analyses, as the standard error for treatment difference per centre could not be obtained as input variables for 'metacont( )'. Performance of six models in scenario 9 was similar to that in scenarios 7 and 8, although point estimates from all models appeared to be marginally biased toward the null (Table 8). Estimates from patient-level models were more precise and closer to 0.230, the best linear unbiased estimate of standard error based on the simulation model. Once again, the standard error was slightly biased upward in Model A and marginally biased downward in Model D. This resulted in wider and conservative interval estimates from Model A and slightly narrower intervals from Model D. Models B and C performed comparably, probably because on average only three centres each containing one patient were dropped from Model B, which did not affect the variance estimation much. Models C and D achieved convergence for all 1000 simulations in this scenario.

**Table 8:** Properties of point and 95% interval estimates calculated from Models A - E based on 1000 simulated datasets in scenario 9 - unbalanced, 46 centres, same centre composition as the COMPETE II trial.

	SCENARIO 9				
**Model**	**Mean effect** **(SD)**	**Ave**. **SE**	**MSE**	**Cover**. **of CI**	**Power**

ICC = 0.125					

A	1.254 (0.236)	0.249	0.056	0.965	0.999

B	1.253 (0.240)	0.236	0.058	0.952	1

C	1.253 (0.237)	0.235	0.056	0.949	0.999

D	1.253 (0.237)	0.230	0.056	0.944	0.999

E-1	1.256 (0.405)	0.207	0.165	0.787	0.991

E-2	1.257 (0.270)	0.261	0.073	0.935	0.995

SD: empirical standard deviation; Ave. SE: average estimated SE; MSE: mean squared error; Cover. of CI: coverage proportion of 95% confidence interval; ICC: intraclass (intracentre) correlation

## Discussion

In this paper, we investigated six modelling strategies in a Frequentist framework to study the effect of an experimental treatment compared to the control treatment in the context of multicentre RCTs with a continuous outcome. We focused on three designs with equal or varying centre sizes and a treatment allocation ratio of 1:1 in the absence of treatment by centre interaction. Results of this simulation study showed that, when the proportion of patients allocated to the experimental treatment was the same in each centre or subject to chance imbalance only, models using patient-level and centre-level data yielded unbiased point estimates of treatment effect across a wide spectrum of ICC values. Ignoring stratification by centre or within-centre correlation did not bias the estimated treatment effects even when ICC was large. In fact, Parzen et al showed that mathematically the usual two-sample t-test, naively assuming independent observations of the response within centre was asymptotically unbiased in this context [30].

The simulation study also indicated that these models produced different standard errors of ${\hat{\beta }}_{1}$, and the properties of interval estimates were affected by several factors: whether and how centre effects were incorporated in analysis, the combination of centre size and number of participating centres, and the level of non-orthogonality of the observed data. Treating centre as a random intercept resulted in the most precise estimate, and nominal values of coverage and power were attained in all circumstances. The fixed-effects model had extremely similar performance compared with the mixed-effects model in balanced design, but was slightly less efficient when the number of centres was large (J > 20) in an unbalanced design. Pickering and Weatherall observed the same pattern in their simulation study comparing three patient-level models with small ICC values [7]. The GEE model using information sandwich covariance method tended to underestimate the standard error across centre effects when the sample of centres was small, a property noticed by researchers [20,31]. This resulted in higher statistical power. That is, the treatment effect estimate was more likely to be significant with a smaller standard error, but was associated with a lower coverage of the conference interval. Marray et al suggested that at least 40 centres should be used to ensure reliable estimate of standard error in the context of cluster randomized trials [32]. Our simulation results suggested that such cut value was also applicable to multicentre RCTs. Failure to control for centre effects in any form resulted in inflation of standard error, falsely high interval coverage and sizable drop of power, as ICC increased. Parzen et al quantified the impact of correlation among observations within centre on the variance of ${\hat{\beta }}_{1}$ in Model A as 1/(1-ICC) [30]. Alternatively, one may consider a variant of robust variance estimation or a GEE model with an independent working correlation to control for the impact of ICC on variance estimation using t-test. Centre-level models generally produced larger standard errors, lower coverage or power than the patient-level models. Centre-level random-effects model incorporated variability of the treatment effect over centres, and was not a fair comparator to other models. Interestingly, this model seemed to fare better than the centre-level fixed-effects model in terms of precision and coverage even though the simulated datasets contained no treatment by centre interaction. Despite that the random-effects centre-level model may be a reasonable alternative for patient-level models when the number of patients per centre is large (≥30), centre-level models cannot adjust for patient-level covariates, a potential fatal drawback in the presence of patient prognostic imbalance.

Statisticians have different viewpoints on treating centre effects and treatment by centre interaction as fixed or random effects when analyzing multicentre RCTs [12,13,21,33]. Our simulation results demonstrated the advantage of treating centres as random intercepts in the absence of treatment by centre interaction. When many centres enrol a few patients and allocation is unbalanced, the random intercept models can give more precise estimates of the treatment effect than the fixed intercept models, because they recover inter-centre information in unbalanced situations. For instance, in a multicentre RCT consisting of 45 centres each recruiting 4 patients, the empirical variance of the estimator of the treatment effect resulting from the fixed-effects model was 24.8% and 26.0% greater than that from the random-effects model when the ICC was 0.01 and 0.05, respectively. In the sentence alluded to, we need to compare the empirical variance of 0.162^2 ^with the value of 0.145^2 ^for ICC = 0.01, and 0.174^2 ^to 0.155^2 ^for ICC = 0.05 (Table 6 scenario 4). We therefore take the same position as Grizzle [33] and Agresti and Hartzel [12] that, "Although the clinics are not randomly chosen, the assumption of random clinic effect will result in tests and confidence intervals that better capture the variability inherent in the system more realistically than clinical effects are considered fixed".

Our results have some implications for the design of multicentre RCTs in the absence of treatment by centre interaction. First, regardless of the pre-determined allocation ratio, permutated block randomization (of relatively small block sizes) should be used to maintain approximate balance or orthogonality (i.e. same treatment allocation proportion across centres [7]) between treatments and centres, so that their individual effects can be evaluated independently. Variable block sizes can be used to strengthen allocation concealment. Second, for a given sample size, the number of patients randomized in majority of centres should be sufficiently large to ensure reliable estimate of within-centre variation. Third, it is essential for investigators to obtain a rough estimate of ICC for within-centre responses, through literature review or a pilot study. To reach nominal power of 80% or 90% (in the absence of clustering), centre effects should be taken into consideration in sample size assessment. When centre effects are included without treatment by centre interaction, the analysis becomes more powerful than a two-sample t-test. One method to assess sample size is to start with a two sample t-test for continuous outcomes (ignoring centre effect) then multiple the original estimated error variance by an variation inflation factor of 1/(1-ICC). This factor would have the effect of increasing the required sample size. Ignoring centre effects results in the larger sample size in the absence of interaction. Sample size determined using information sandwich covariance of GEE model could lead to slight loss of power, when the number of centres is small (≥40) and no proper adjustment is done. Lastly, there is no particular reason to require equal numbers of patients being enrolled in all participating centres and this is seldom the case in practice. Throughout the simulations, we observed similar results for studies of equal and varying centre sizes. In the study, we considered three scenarios representing the particular centre composition of the COMPETE II trial. For discussion on potential impact of enrolment patterns on the point and interval estimates of treatment effect, readers can refer to the publications on random enrolment verse determined enrolment, and relative efficiency between equal and unequal cluster sizes in the reference list [34,35].

The current ICH E9 guideline recommends that researchers investigate treatment effect using a model that allows for centre differences in the absence of treatment by centre interaction [1]. However, it is implausible or impractical to include centre effects in statistical modelling or stratify randomization by centre, when it is anticipated from the start that trials may have very few subjects per centre. As it is acknowledged in the document, these recommendations are based on fixed-effects models. Mixed-effects models on the other hand may also be used to explore the centre and centre by interaction effects, especially when the number of centres is large [1]. Our simulation results indicated that when a considerable number of centres contains only a few patients, adjusting for centre as a fixed effect may lead to reduced precision (depending on distribution of patients between arms) compared with the naïve unadjusted analysis. Our work complements the ICH E9 guideline, by studying the impact of intraclass correlation on the assessment of treatment effects - a challenge that is seldom discussed, although routinely faced by investigators in reality. Our investigation suggests that, (1) ignoring centre effects completely may cause substantial overestimation of the standard error, faulty increase of coverage of the confidence interval and reduction of power; and (2) mixed-effects models and GEE models, if employed appropriately, can produce accurate and precise effect estimates, regardless of the degree of clustering. We recommend consider these methods in developing future guidelines.

When the number of patients per centre is very small, it is not practical to include centre as a fixed effect to analyze patient-level data, as centre effects cannot be reliably estimated, and precision of the treatment effect will be compromised. In fact for extremely small centres, all patients may be allocated to the same treatment group, and such centres will be ignored by the fixed-effects model [36-39]. The alternatives include collapsing all centres to perform a two-sample t-test, collapsing smaller centres to create an artificial centre and treating it as a fixed effect, and exploring other models discussed above. The mixed-effects model utilizes small centres more efficiently by "borrowing" information from larger centres. The GEE approach models the average treatment difference across all centres and adjusts for centre effects through a uniform correlation structure. This is an intuitively more efficient model which unfortunately does not always converge when the number of patients per centre was highly variable (simulation scenarios 7 and 8). In the current study, non-convergence problems were more likely to arise for very small or large ICC values (less than 0.1 or greater than 0.4 for block size 2 or 4) due to non-positive definite working correlation matrices, and the frequency could be as big as 10% after 2000 iterations. Conversely, convergence problems did not occur for the mixed-effects models in any scenarios. Our results show that analysis of trials consisting of very small centres (i.e. those containing less than 2 patients per arm) using centre-level models may not be an optimal strategy, because the within-centre standard deviation of treatment difference cannot be estimated for such centres, and consequently these very small centres are excluded from the analysis.

Results of two large empirical studies and one systematic review of cluster RCTs in primary care clinics suggested that most ICC values on physical, functional and social measures were less than 0.10 [26-28]. The estimated ICC in the COMPETE II trial using GEE and linear mixed-effects model, on the other hand, was 0.124 and 0.138, respectively. We chose to include rare yet possible large ICC values (0-0.75) in this simulation to examine the overall trend of model performance by ICC, and for the purpose of completeness and generalizability. Readers should anticipate the ICC values likely to emerge from their studies when interpreting these results. Throughout the work, we quantified correlation among subjects within centre using ICC, the most commonly used concept to assess clustering in biomedical literature. As indicated in previous sections, ICC reflects the interplay of two variance components in multicentre data: the between-centre variance and within-centre variance. These variance components are relatively easy to interpret for analysis of continuous outcomes using linear models. For analysis of binary or time-to-event data from multicentre trials using generalized mixed and frailty models, interpretation of centre heterogeneity can present challenges because random effects are linked to the outcome via nonlinear functions [40]. Reparameterization of the probability density function may be used to assess the impact of within- and between-centre variance. Interested readers can refer to Duchateau and Janssen [40] for more details.

A major limitation of the study is that it did not address model performance when the treatment by centre interaction exists. The interactions may be due to different patient populations or variable standard of care. Interested readers may read Moerbeek et al [6] for formulas of variance of ${\hat{\beta }}_{1}$ in different models and Jones et al [14] for simulation results. Future studies addressing interaction effects in multicentre RCTs are needed. Datasets in the current paper were generated based on a moderate treatment effect reflected by the standardized mean difference between the treatment and control group. More or less prominent treatment effects are also likely to occur in clinical studies and similar findings are expected. The current study investigated on continuous outcomes in two groups from a Frequentist perspective. The models discussed above can be naturally extended to compare three or more treatments. Agresti and Hartzel [12] surveyed different methods to evaluate treatments for binary outcomes in multicentre RCTs. Non-parametric approaches and Bayesian methods are also available to obtain treatment contrast. Interested readers can refer to Aitkin [41], Gould [11], Smith et al [42], Legrand et al [16], and Louis [43], to name a few.

## Conclusions

We used simulations to investigate the performance of six statistical approaches that have been advocated to analyze continuous outcomes in multicentre RCTs. Our simulation study showed that all six models produced unbiased estimates of treatment effect in individual patient randomization multicentre trials. Adjusting for centre as random effects resulted in more efficient effect estimates in all scenarios over a wide spectrum of ICC values and various centre compositions. Fixed-effects model performed comparably to the mixed-effects model under most circumstances but lost efficiency when many centres contained a relatively small number of patients. The GEE model underestimated standard error of the effect estimates when a small number of centres were involved, and did not always converge when the centre size was variable for very large or small ICC values. Two-sample t-test severely overestimated standard error given moderate to large ICC values. The relative efficiencyof statistical modelling of treatment contrasts was also affected by ICC, distribution of patient enrolment, centre size and the number of centres.

## Competing interests

The authors declare that they have no competing interests.

## Authors' contributions

RC participated in the design of the study, simulation, analysis and interpretation of data, and drafting and revision of the manuscript. LT contributed to the conception and design of the study, interpretation of data and revision of the manuscript. JM contributed to the design of the study and revision of the manuscript. AH contributed to acquisition of data and critical revision of the manuscript. EP and PJD advised on critical revision of the manuscript for important intellectual content. All authors have read and approved the final manuscript.

## Pre-publication history

The pre-publication history for this paper can be accessed here:

http://www.biomedcentral.com/1471-2288/11/21/prepub
